# Co-designed mini-games for children with visual impairment: a pilot study on their usability

**DOI:** 10.1007/s11042-022-13665-7

**Published:** 2022-09-09

**Authors:** Tiziana Battistin, Nadir Dalla Pozza, Silvia Trentin, Giovanni Volpin, Andrea Franceschini, Antonio Rodà

**Affiliations:** 1 Robert Hollman Foundation, via Siena 1, Padova, 35143 Italy; 2 Department of Neuroscience and Rehabilitation, University of Ferrara, via L. Borsari, 46, Ferrara, 44121 Italy; 3 Department of Information Engineering, University of Padova, via Gradenigo 6b, Padova, 35131 Italy; 4 Department of Computer Science and Technology, University of Cambridge, 15 J J Thomson Avenue, Cambridge, CB3 0FD UK

**Keywords:** Serious games, Large scale interactive environment, Children with visual impairment, Human-computer interaction

## Abstract

Digital games aimed at improving cognitive and/or motor-sensory skills need to be carefully designed to take into account the characteristics and needs of particular categories of users. Several novel mini-games explicitly aimed at children with visual impairment (VI) were co-designed by a multidisciplinary team which involved computer engineers and a therapy team from the Robert Hollman Foundation (Padova, Italy). These games are played by children moving within a large-scale interactive environment – i.e., a floor portion placed under a motion capture system capable of tracking one or more people – with the game linking the players movements to the audio and visual output to produce meaningful interactions. We report on a pilot study of the usability of the system involving 11 children with VI. The results allowed us to improve the system and to define a set of guidelines useful for designers and developers of similar systems.

**Supplementary Information:**

The online version contains supplementary material available at 10.1007/s11042-022-13665-7.

## Introduction

Many studies show that, if well designed, digital games can improve many specific skills, including cognitive, emotional, and sensory-motor skills.

In this context, the See-Sound project aims at developing and testing some mini-games suitable for the training of children with visual impairment (VI), in the range of age between 3 and 8 years. Using a co-design approach, together with the Robert Hollman Foundation (RHF), we designed three mini-games for children with different visual impairments [28]. We tried these mini-games with a small cohort of children with VI, aged 3 to 8 years, to test the games’ usability and acceptance. The Robert Hollman Foundation is a private non-profit Dutch Organization offering free advice and support for the development of children with visual impairment, and their families, since 1979.

A visual disorder, occurring in the early years, affects the comprehensive neurodevelopment of the child [26]. Specifically, children with VI often show delays in emotive-affective, psychomotor, and neuropsychological development, as well as in the learning process. In 1977, Fraiberg defined vision as the “central agency of sensorimotor adaptation”, a sort of “synthesizer of experience” [13]. Eyesight is considered the main sense, which sets the prerequisites for future social relations, thus enabling the establishment of affective relationships. Due to the reduction of the visual information from the surrounding environment, children with VI may show a lack of social and motor initiative, with psychomotor delays and specific difficulties in spatial navigation and other spatial skills [9]. A multidisciplinary and multidimensional therapeutic approach is required because of the impact of the VI on the overall development of the child [24]. The shared aim of the RHF rehabilitation team is to promote a social and motor initiative of children with VI towards the surrounding environment through a customized care path for each child, shared with their family and according to their needs. Play is the main tool through which it is possible to create a therapeutic relationship with the child, allowing them to express themselves, to learn new cognitive, social, and sensory-motor skills, and to become aware of their own potential. Furthermore, by playing, children gradually improve and mature their proprioception, their body awareness, and their spatial orientation. Children with VI sometimes show solitary play, they need longer time to explore toys, and they may have difficulty in understanding social communication and cooperative play. Therefore, it is crucial to create the most facilitating conditions to help them to better express themselves through play. This can be achieved by giving them time to understand a new game or to adapt to a new situation, verbally anticipating something that they may find hard to predict (e.g., the sudden arrival of a ball), physically guiding them to explore the toy, maintaining a stable environmental layout, using bright and contrasting colours, and introducing multi-modal feedback, such as tactile and/or acoustic stimuli. The creation of facilitating environments and proposals is fundamental to support and favour the emergence of new competencies and to promote fun, motivation, and a proactive motor and social initiative.

In the See-Sound project, we are exploring the potential of multi-modal interaction, by using full-body movement and proprioception as communication channels, complementary to audio and video, for accessing digital games and pursue learning and rehabilitation goals that are specific to children with VI. We developed a large-scale interactive environment running several mini-games and an interface to allow therapists to modify most of the game parameters, to easily adapt the games to the specific needs of the children. Other applications of this environment are listed in [16, 18–20]. The goal is to help children by providing them with an exciting and educational environment, but also to foster fun and interaction with the outside world. Our large-scale interactive environment comprises an overhead motion tracking system and video projector, and the floor area within the motion tracking system’s range is used as a projection surface and interaction space. A computer processes the data coming from the motion sensors and outputs the coordinates of one or more people inside the active area, allowing us to link the users’ positions to interactive audio and graphics.

The mini-games were already described the first time in Rodà et al. [28]. The main novel contribution of this paper is to present the results of a pilot study in which these mini-games were used by children with VI in a real therapeutic context. In particular, we are interested in understanding: a) if these kind of games, requiring full-body movements and visual attention, are well received by children with VI, b) to what extent these activities can be integrated in the rehabilitation path of children with VI, and c) what are the technical requirements to design large-scale interactive environments for children with VI.

After a review of the main related work (Section 2), in Section 3 we report on the design process of the games and the list of requirements that resulted in the final implementation. The results of the pilot study are summarized in Section 4 and discussed in Section 5, along with a set of guidelines aimed at helping the design and development of digital games for children with VI.

## Related work

Assistive technologies for people with visual impairment or blindness are growing fast, providing useful tools to support daily activities and to improve social inclusion. Most of these technologies are mainly focused on helping people with severe visual impairment to navigate and avoid obstacles, or on providing them assistance to recognize their surrounding objects. One example is a prototype that enables visually impaired or blind people to move autonomously, and helps them to recognize multiple objects in public indoor environments, using a reasonably-sized integrated device placed on the chest [22]. Another example is the “Canetroller”, a haptic cane controller which simulates the white cane interactions enabling people with visual impairment to navigate a virtual reality environment by transferring their cane skills into the virtual world [31]. Unfortunately, as is the case with these two examples, most of these technologies are designed for adults with already developed cognitive and spatial motor skills. Digital serious games are a relatively recent technology that can be used used for learning and rehabilitation in a playful environment. The pedagogic component of these games is the key factor in making them serious, although their entertainment component, which uses storytelling, art, and software elements of the game, is the most immediately prominent aspect.

Serious games are also used to help people with various kinds of disabilities, because they serve to train and monitor many aspects of the person, such as coordination, memory development, communication skills, and socialization. For example, a visual acuity test for children can be disguised as a very amusing game [6]. Serious games may be very good for children, because they allow an accurate assessment during play, and at the same time they can provide rehabilitation and telerehabilitation programs that can last for a long time, being attractive for the very young players [27]. Even though serious games are considered an effective intervention in rehabilitation, these games must comply with adaptability and configurability criteria to adapt to the patient skills, in order to best support therapy. For this reason, designers must include the possibility of customizing the game on the different needs of the patients [25]. Designers must also address accessibility in the case of involvement of people with VI: the visual component, which is typical of video-games, must be replaced or complemented by other modalities such as sound and haptics. Feedback is very important to the user with visual impairment, so the games must be designed around these three challenges: difficulty of using interaction devices, difficulty of receiving feedback, and difficulty of identifying the set of answers [30]. Types of feedback often used by accessible games are text-to-speech (to read out interface elements), audio metaphors (intuitive audio feedback that helps to perceive environment-specific sounds and game events, such as opening a door or the success or failure of a game action) in association with 3D audio (spatial audio reproduction technique to help identify the position of sound sources, to locate game elements, and to serve as orientation in the scenario), and tactile feedback to provide responses when the user interacts with interface elements (e.g., vibration when the screen is touched) and/or some game elements (e.g., vibration when the character bumps into an obstacle)^1^.

With regard to visual impairment, there are some examples of serious games that use these components in order to achieve different aims. “HelpMe!” [10] is a serious game with the objective to improve the rehabilitation process for children with Cerebral Visual Impairment (CVI), using a system which can adapt the exercises to each child and to their improvements. The system also integrates an eye tracker system to record the child’s eyes movement during the rehabilitation program to correctly measure their performances and their capability to watch and touch a moving object at the same time. This helps the professionals to understand if the training process has a positive result, providing them with a precise way to measure the performance of the children during the whole rehabilitation training programs. “Virtual stage!” [11] is a tablet-based musical game which uses the audio modality as the main component. It stages a virtual musical band with different instruments, and the player moves an avatar around the virtual stage searching for a specific instrument. The game has been developed using binaural audio techniques (3D audio) for the instruments’ sounds to help the player locate the instruments in the virtual space, and to guide them during their interaction with the tablet. “Blindside” and “The Nightjar” [23] are two further examples of games designed around the idea of using primarily auditory cues. The first one is a role-playing horror-adventure game which makes extensive use of 3D audio as a gameplay element. The game consists in navigating around a city to reach a point of safety, by relying solely on audio cues. Although “Blindside” is designed for entertainment purposes, it advertises itself as a serious game with a strong emphasis on accessibility, with a focus on the community of people with visual impairment. “The Nightjar” is a role-playing mobile game where players only use spatial references given by auditory feedback to navigate within a spacecraft called “Nightjar,” simulating the effect of blindness. The game offers a simple user interface to control the avatar by tapping the on-screen buttons. “The Nightjar” does not promote itself as a serious game but it delivers subliminal messages that are found in edutainment. Unfortunately, both games are unavailable for download at the time of writing. A similar game to the previous ones, and designed specifically (and fully accessible) for people with VI, is “A Bling Legend” [29]. This is a mobile action-adventure game that only uses 3D sounds and no visuals to guide the player inside the story’s chapters. The smartphone’s screen or computer keyboard are used to move the character, to fight, and to interact with the menu. This helps hearing stimulation and hear-hand coordination. “Eda Play” [29] is a collection of applications for tablet devices designed under supervision of experts in the field of vision stimulation and early intervention for children with special needs. These applications are tailor-made for children with visual impairment and multiple disorders, to help them train their vision and fine motor skills thanks to illustrations using comprehensible shapes, boldly colored, large size pictures with high figure/ground contrast, and tasks ranging from simply watching the action and screen-touching, to more complex hand-eye coordination tasks. Another major issue with digital serious games for people with VI is the interaction between the human and the video game, which often has to be structured appropriately. A number of different approaches for human-computer interaction are explained in Mahmud et al. [15]. An example is “BlindPAD” [14], which is based on a different approach, because it mainly uses haptics as the main mode of interaction between the player and the game. In this game, a tactile pin-matrix display made of hundreds of “taxels” (i.e., tactile equivalent of pixels) is used to interface the player with a set of games based on maps and geometric shapes perceived through their fingers. During the TiM project [2], a project with the overall aim to provide young children with VI multimedia computer games they can access independently (without the assistance of a sighted person), other tactile games have been developed. One of these was an accessible version of “Reader rabbit’s Toddler” , an educational video game where the player only has to roll the mouse over the hotspots to interact with the game. Instead of using the mouse, the children can feel tactile buttons on a tactile board and drive the game using these [3]. Another example is “FindIt” [4], a very simple audio/tactile discovery and matching game for very young children, or children with additional disabilities, where the player must connect sounds or audio clues associated to items with images on the screen or tactile information on a tactile board.

Although all these games show good potential to address the specific needs of children with VI, none include full-body movement. As shown in some pilot experiments [16, 17, 21], a full-body approach with people with VI can be highly effective.

## The mini-games

The three mini-games^2^ presented in this article were designed following a co-design approach inspired by Design Thinking (DT) [8].

**Fig. 1:**
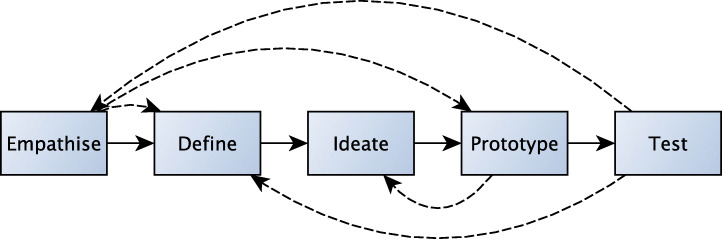
The five steps of the Design Thinking methodology

This approach is based on five steps (see Fig. 1): empathise, define, ideate, prototype and test. It is important to note that the five stages should be understood as different modes contributing to a project, with many interactions between them, rather than sequential steps. The design process involved a team comprising therapists from the Robert Hollman Foundation and computer engineers from the Department of Engineering of the University of Padova.

To foster empathising, all the meetings except for one were held at the rehabilitation center of the Foundation. In this way, the computer engineers gained a deeper personal understanding of the issues involved, by acquiring first-hand experience of the physical environment, which is particularly important since the games are actually situated on the floor of a room. The engineers got to meet the final users, and observe the various learning and rehabilitation activities provided by the Foundation. Moreover, to promote greater awareness of the difficulties of a child with severe visual impairment, the engineers were offered a sensory reduction experience using glasses that allowed them to simulate a reduction in vision which can be present in many visual pathologies [12].

During the first few meetings, the team defined the following problem statements, for which the designers tried to find a solution in the form of a digital game to support therapy:

- P1: children with VI struggle to interact with small and moving objects, such as a ball or a balloon. The game will present digital objects with adequate dimensions and dynamics.
- P2: children with VI, especially those with early onset loss of sight, have difficulties in spatial orientation. The game will help to encourage the development of spatial orientation skills through interaction with visual and/or acoustic stimuli.
- P3: children with VI sometimes have low proprioceptive awareness, assuming incorrect postures from a musculoskeletal point of view. Often these incorrect postures are functional to the use of their residual visual function. The game will help to develop body awareness by having different visual/sound feedback based on body movements.
- P4: children with VI usually differ greatly by type and intensity of the visual impairment. The system must be easily adaptable to the needs of each child, by allowing the operator to customize the experience with the child by introducing, eliminating, or modulating elements of the scenario. To meet this need, the system will have some tools to easily set various parameters – e.g., background colour, colour and size of the objects, speed of the objects, sounds, and so on.
- P5: children with VI often have to follow long and demanding rehabilitation programs. The system will have a playful component, exploiting the engagement mechanisms typical of gamification.
- P6: children with VI usually have few opportunities to work in groups. The system will help to improve basic relationship mechanisms through exchange games and turn-based games.

Given these objectives, we designed a set of mini-games for a large-scale interactive environment. The choice of this user interface results from the following considerations: a) the need for spatial orientation training requires full-body movements in a large environment, at least inside a room; b) by projecting the visual objects on a floor, we can magnify them as required by the specific impairment; c) in comparison to completing tasks on a screen, the interaction with a physical environment, such as the floor of a room, can potentially improve the transfer of acquired skills to the real world; d) using a large environment facilitates interaction with the therapists, and socialization.

Following the iterative approach typical of design thinking, several prototypes of the games were developed using Processing^3^, a Java-based framework that is particularly well suited for fast prototyping. All the parameters and characteristics of the games have been decided in collaboration with the operators of the RHF, and specific meetings were held in which all the variable parts of the games were decided, such as shapes, colours, speed, size and number of objects, etc. The RHF operators needed to be autonomous in configuring some of these parameters, with the aim of customizing each game for each child’s different set of abilities.

**Fig. 2:**
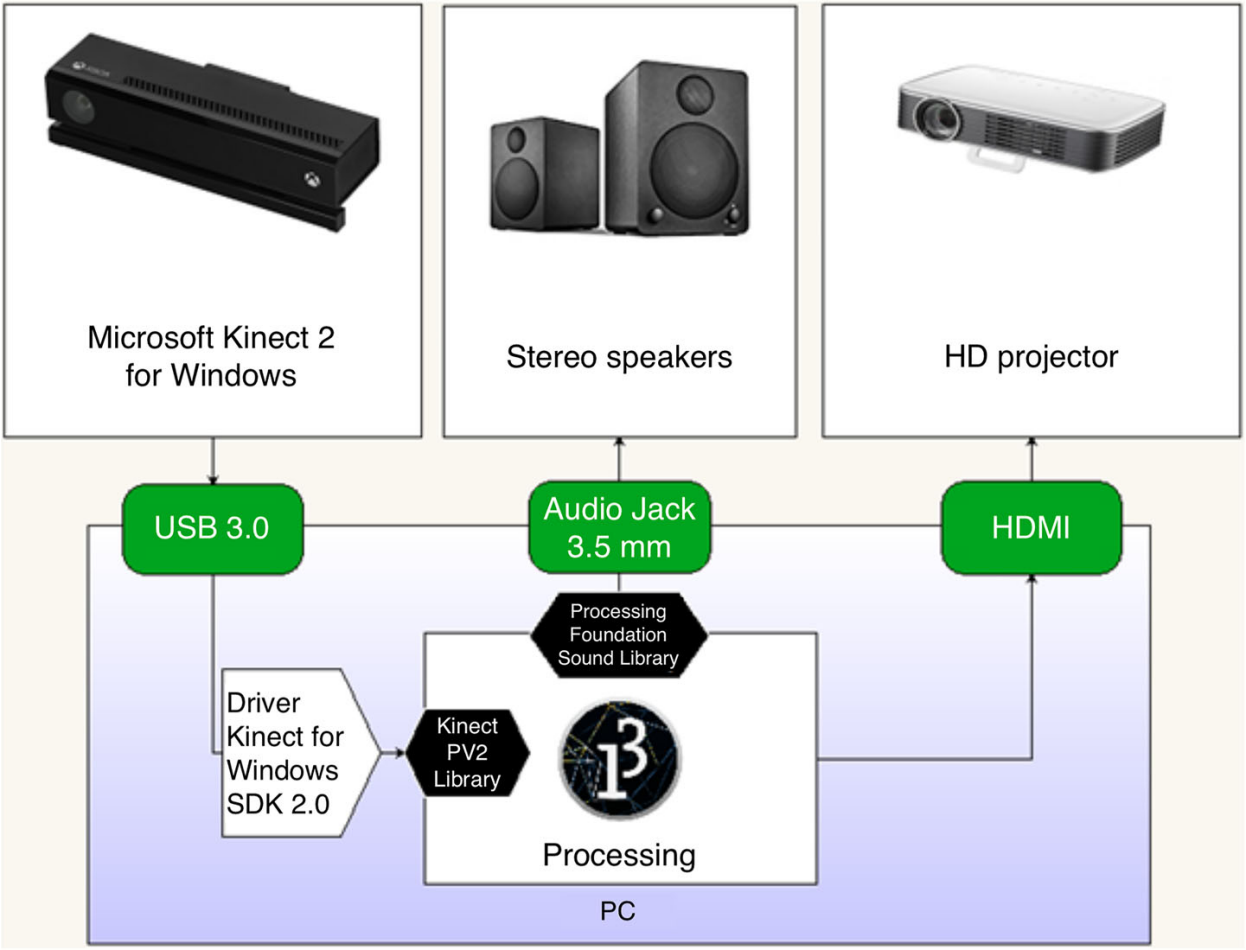
Software-hardware architecture

Figure 2 shows the architecture of the large-scale interactive environment used for the games: the system employs a PC, a motion tracking device (Microsoft Kinect 2 for Windows) connected to the PC, and a video projector. Both the sensor and the projector are mounted on the ceiling pointing downwards, perpendicular to the floor. All the software components were developed using Processing, including an application (a “*sketch*” in Processing lingo) to track people from a top-down perspective. The moving centroids of the people inside the field of view of the motion sensor are used as inputs in each game. The visual output is projected on the floor in a 3.5 × 2m game field using a single projector mounted on the ceiling.

The floor was covered with light gray linoleum to improve the quality of the projection, and the contrast in particular, which is a very important aspect for children with VI.

In the following sections, we present the three mini-games that we designed and implemented. To improve the game usability and final user experience, a single launch application has been developed, where each game is represented by a coloured rectangle bearing its name so that it can be easily identified and launched.

### Bubbles

This game surrounds the player with coloured, moving bubbles. The player’s goal is to burst several bubbles as quickly as possible by stepping on them.

The *Bubbles* game was designed with the following objectives, related to the problem statements P1, P3, P4, P5 (see Section 3): i) being fun; ii)training coordination (visuo-motor coordination in particular), reaction time, selective attention, and colour recognition; and iii) improving the ability to predict a rectilinear trajectory and to plan related movements.

**Fig. 3:**
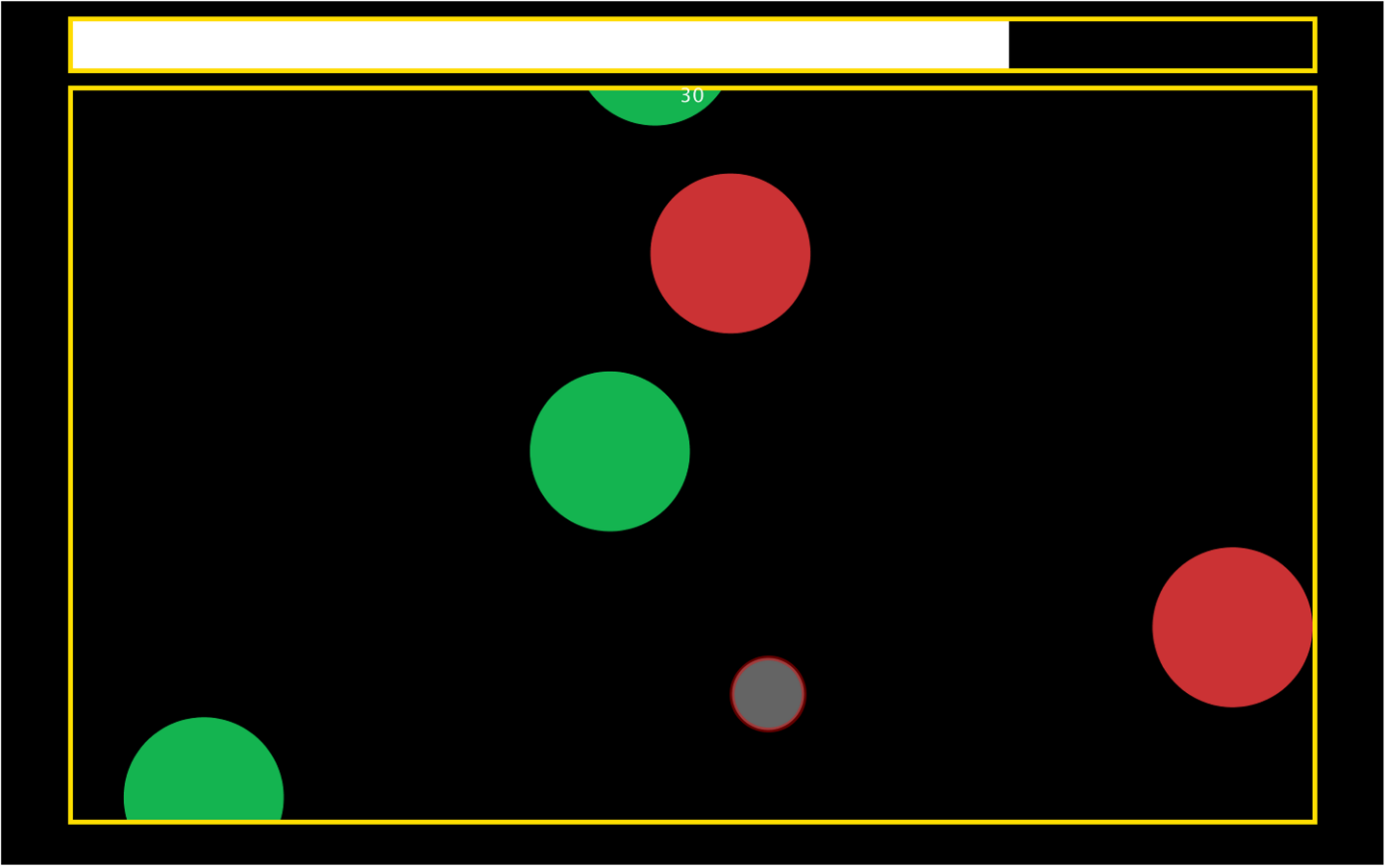
The playground of *Bubbles*

The game has a rectangular playground, with a monochrome background (see Fig. 3) in which moving bubbles appear, coming in from the borders. During game play, a horizontal bar on the top of the screen slowly decreases to mark the passage of time, and increases by a set amount whenever the player bursts a bubble. The game ends when the bar is fully depleted. This mechanism introduces a penalty for inactivity and stimulates the search of the next bubble. In addition to this, a timer reports the seconds elapsed from the start of the game to give a feedback to the operator. Both the bar and the timer can be disabled, if desired.

**Fig. 4:**
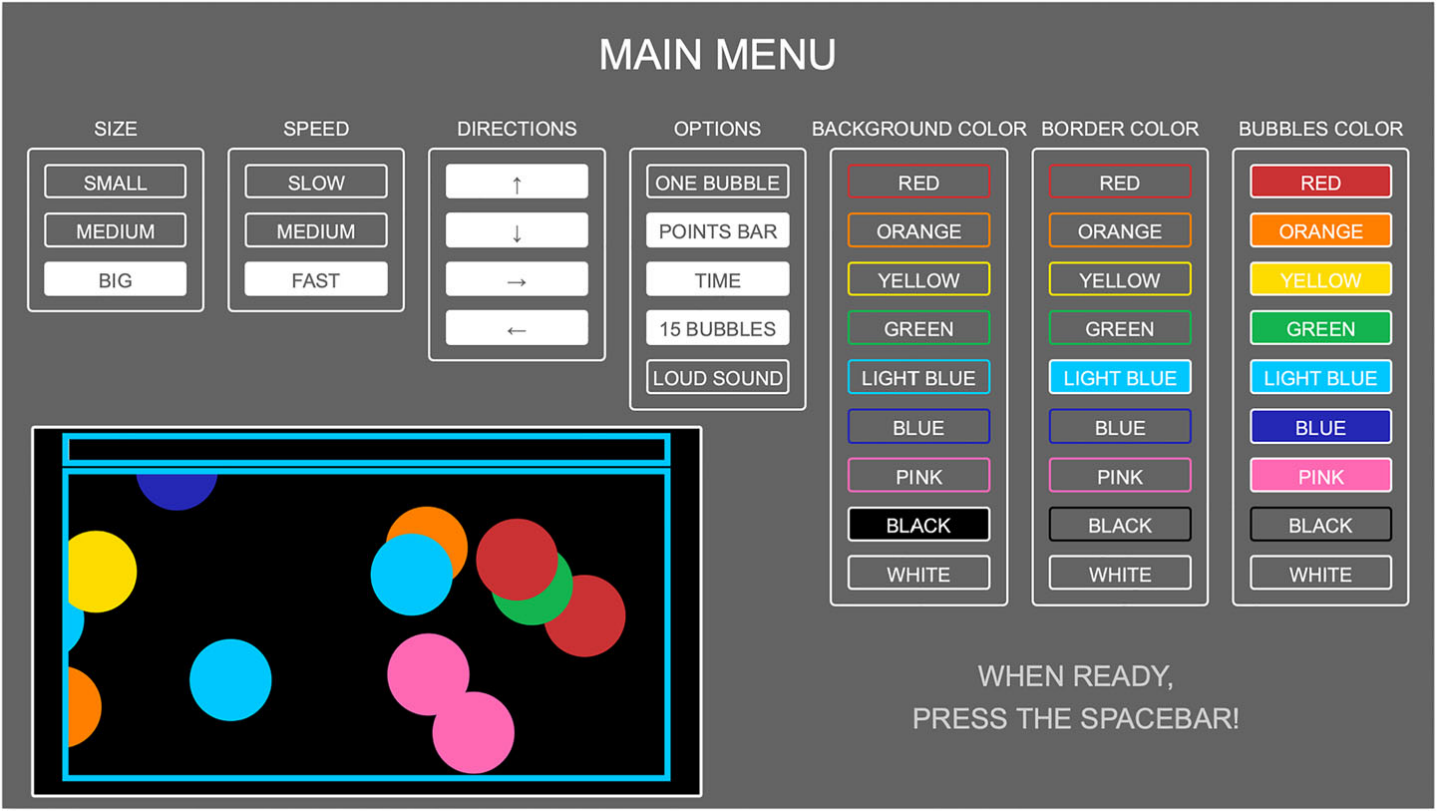
The interface that allows the therapists to setup *Bubbles* parameters, in relation to the specific needs of the children

A comprehensive configuration interface (see Fig. 4) has been developed to allow the therapist to change several parameters, in relation to the specific needs of each child. In particular, it is possible to change the size of the bubbles, their moving speed, the direction of the movement, the sound library, as well as the colours of the bubbles and of the environment. To further ease the configuration, a preview of the final game field is shown to the therapist.

Following specific requests after initial testing, two other game modes were added that modify the standard game mechanics:

1. *One bubble*: only one bubble is generated at a time, requiring the current bubble to be burst before the next one appears; due to the low number of available bubbles, the top bar decreasing behaviour has been highly reduced. This mode is particularly suitable for children that could be confused when a high number of bubbles surrounds them.
2. *Ten bubbles*: The game finishes when ten bubbles have been burst. This is the alternative to the time bar, where no penalties are given for inactivity.

This game also provides a tool to visualise the execution of previous runs, by replaying the position of the children and of all the bubbles during a game. Together with an interface to manage all the recorded games, this is particularly useful for an offline analysis of the children behaviour.

### Sound explorer

**Fig. 5:**
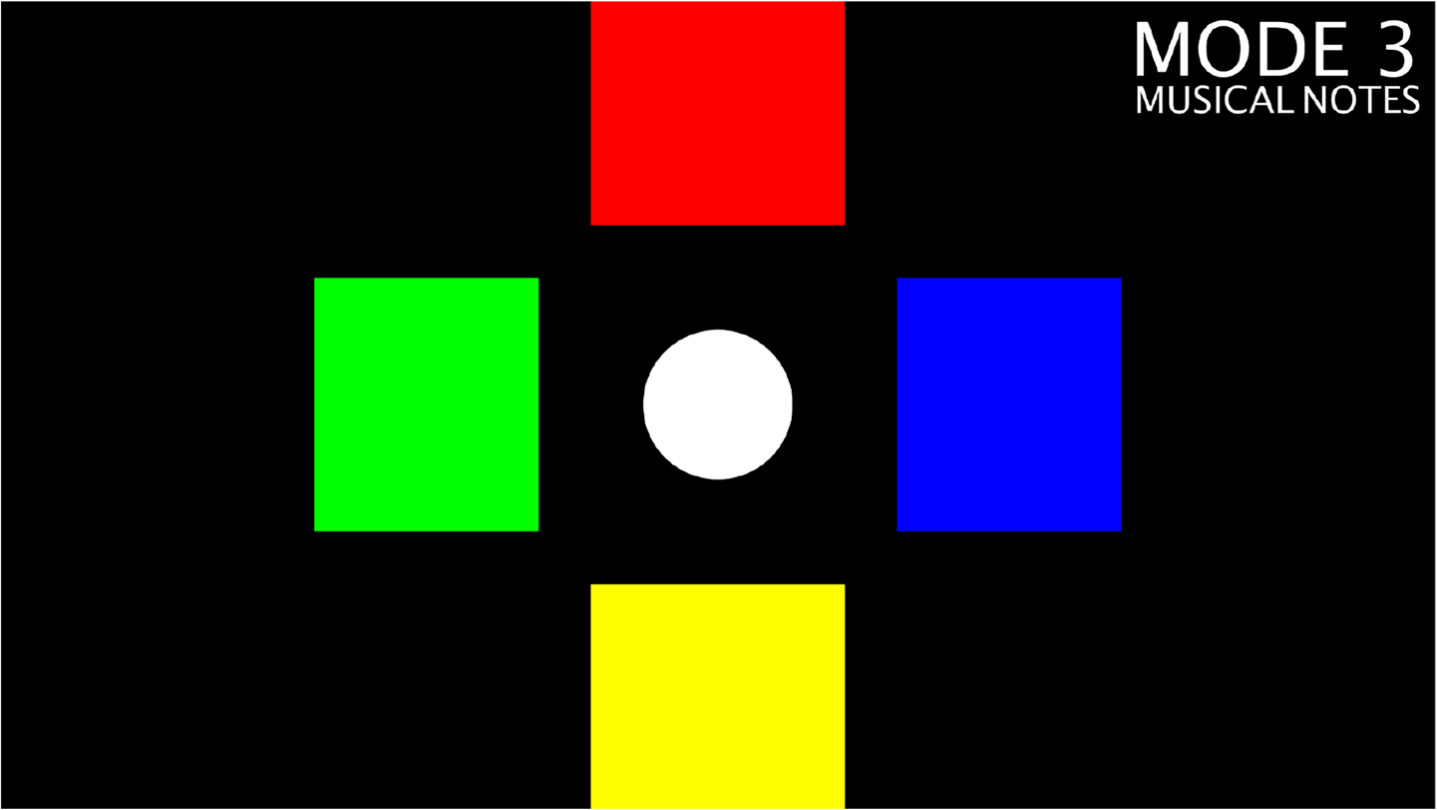
The *Sound explorer* when played in mode 3

The main objective of *Sound explorer* is to look for sounds “placed” on the floor around the player: to listen to a sound, the player must reach its position which is visually marked by a coloured rectangle (see Fig. 5).

This activity was designed to address the problem statement P2, therefore the objective is to develop and improve the ability of understanding and recognizing absolute spatial references. Unlike the other mini-games, *Sound explorer* is also playable by completely blind people.

As spatial orientation training requires a very flexible approach, therapists required that the game should not implement a predetermined game mechanic. Instead, they suggested sort of a sandbox to verbally propose funny activities to the child, such as “find the kitten”. In this case, the pleasant sound that starts when the child reaches the correct position constitutes the reward. To increase the variety of the proposed experiences, the therapist can choose among three sound libraries, namely animal noises, sounds of nature, and musical notes.

This game has four modes:

1. Four coloured rectangles are positioned around the center, always visible and active;
2. As above but with eight rectangles.
3. Four coloured rectangles can be shown and activated by using the arrow keys on the keyboard, to create a path that the player should follow;
4. Four coloured rectangles are shown on the floor, but on the activation of one of them, the activated rectangle disappears for a few seconds after the corresponding sound is emitted.

As in *Bubbles* game, a comprehensive configuration interface was developed to allow the therapists to choose the game mode and the sound library.

### Ping pong

**Fig. 6:**
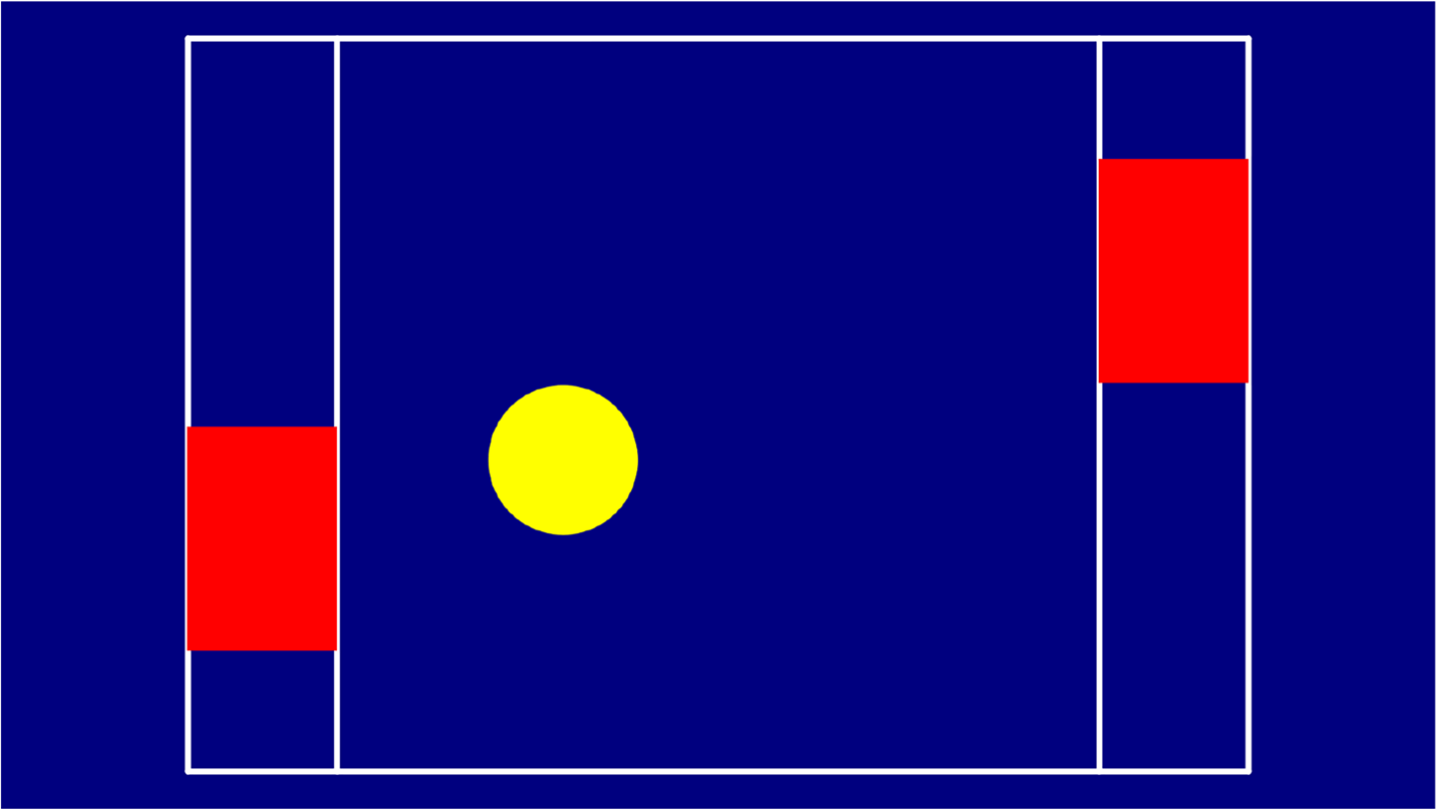
The *Ping pong* game field

*Ping pong* is a remake of an early computer games, recreated in a large scale interactive environment: a simple tennis-like game featuring a field, two paddles – therefore two players – and a ball. Each player controls one paddle by using their body, since the position of the paddle (the red rectangle in Fig. 6) corresponds to the position of the player.

This game responds to the therapy need expressed in the problem statement P6 – i.e., to carry out exchange games – in support of the relationship and expectation of each turn. Moreover, playing with *Ping pong* requires good visual-motor coordination combined with audio feedback generated when the ball hits a paddle or the playground lines.

Similarly to *Bubbles*, the ball is adjustable in size and speed by the therapist using the game menu, depending on the children needs. To facilitate the identification of the game elements, the colors that have been chosen are always of high contrast, as shown in Fig. 6, and all the lines of the game field have a high thickness of about 5 cm. There is also an optional parameter where, after the ball is touched by a paddle, the ball slightly increases its speed: this is an entertaining feature that requires players to be faster and faster as the game keeps going.

## Evaluation

A pilot study, with the participation of 11 children with VI and four therapists of the RHF, was carried out with the aim to evaluate: a) the global satisfaction of the children, and b) whether the approach is suitable for the RHF’s therapists, both in terms of usability and usefulness as a tool inside a therapeutic path.

### Subjects

Participants were recruited among children with visual impairment referring to the RHF. The main inclusion criteria were: age between 3 and 8 years, and confirmed diagnosis of Low Vision. The main exclusion criteria were: presence of a degenerative visual and/or physical pathology, or other associated disabilities. The recruitment was conducted by a member of the research team, who explained the study design to the families. This project was carried out after the approval of the Institutional Board (R14-2021 RHF, Padua, Italy). All the parents gave their informed consent to include their children in the study, and were informed that it was possible to withdraw from the study at any time. In total, 11 children participated in the study. Table 1 summarises their characteristics.

**Table 1:** Sample characteristics

(a) Sample summary, with some statistics.				
Age (years)	Mean	5.68		
	SD	1.77		
Sex	Female	5		
	Male	6		
Diagnosis	Moderate low vision	8		
	Severe low vision	1		
	Blindness, monocular	2		
(b) Details for each child.				
#	Sex	Age	WHO-ICD11 VI	Sessions
			Classification Category	
1	M	5y11m	9.D90.2	2
2	M	6y7m	9.D90.2	2
3	M	2y7m	9.D90.3	4
4	F	8y4m	0.D90.2	3
5	F	5y10m	9.D90.2	1
6	F	5y10m	9.D90.2	3
7	F	7y7m	9.D90.2	1
8	M	5y3m	9.D90.2	4
9	M	7y5m	9.D90.5	4
10	F	5y6m	9.D90.2	3
11	M	3y3m	9.D90.5	2

Participation was less consistent than expected because of the COVID-19 pandemic and other seasonal illnesses, and none of the children participated in all the five sessions. Furthermore, one very young child left the study in the third week because he was not able to adapt to the new proposal, being scared by the lights.

### Materials

**Fig. 7:**
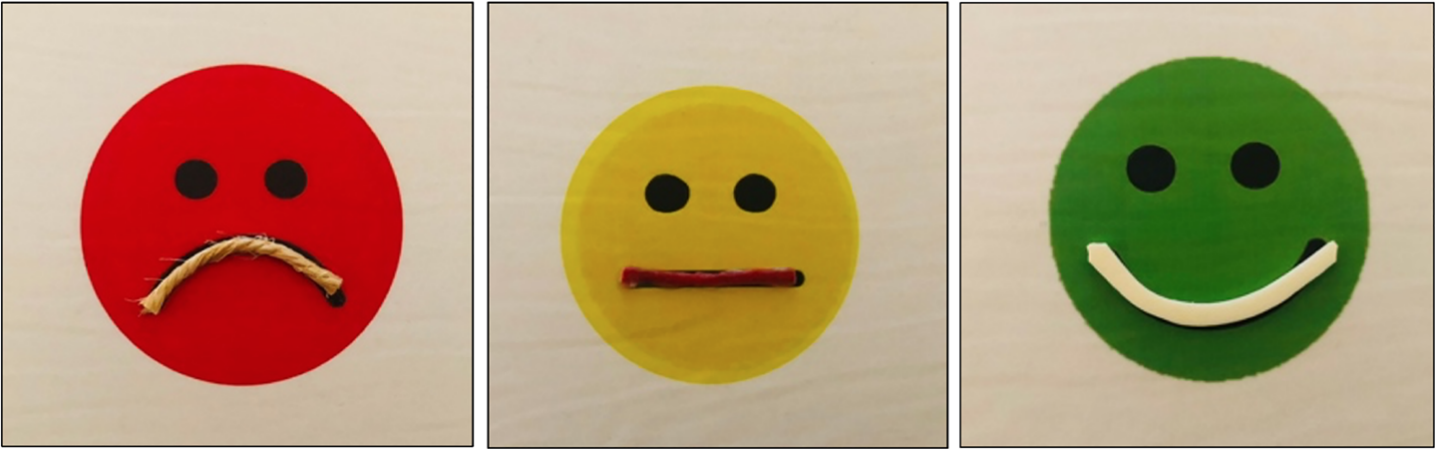
The satisfaction VAS scale

The subjective satisfaction of children was assessed at the end of each session by a visuo-tactile VAS scale, adapted for children with VI (Fig. 7). The VAS scale is an analogue psychometric detection system [1] which allows to collect an individual’s perceptions of a specific situation or experience in a simplified way.

**Fig. 8:**
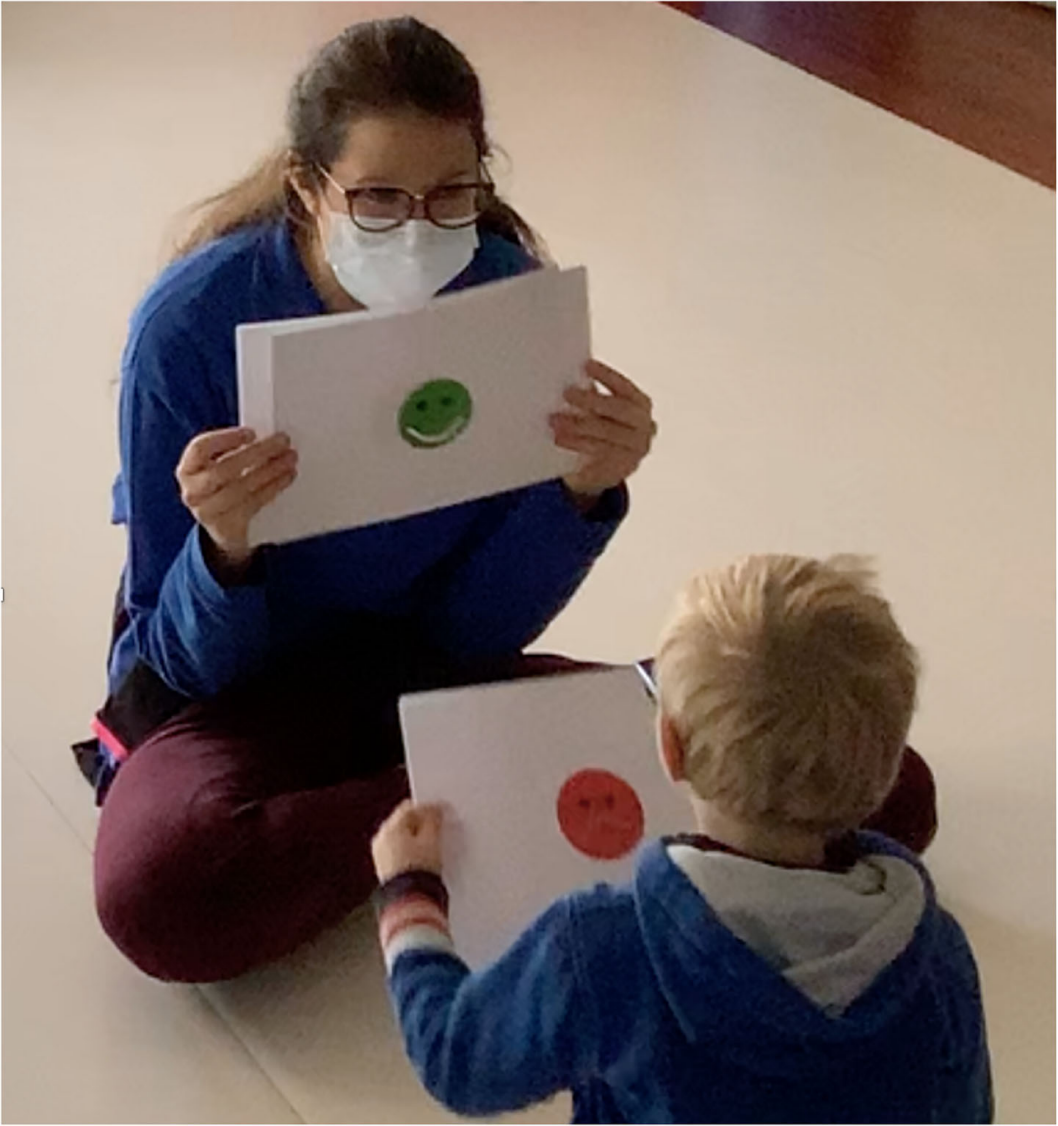
Proposal of the tactile Satisfaction VAS scale to a child

This adapted VAS scale comprised three smileys corresponding to “not at all” for the sad red face, “so and so” for the yellow face, and “very much” for the happy green face. The smileys have bright and contrasting colours which are easily perceivable by children with VI, and a tactile mouth which could be sensed by the children. Touching the faces’ mouths and their orientations (down, straight, or up) could easily help them to understand the corresponding smiley. The presentation of the figure to the children was the same at every session: first, the therapists showed them individually, giving the children time to explore each face both with the visual and the tactile modalities, and then all the three smileys together. These were presented in front of the child, as shown in Fig. 8: the therapist asked the question “Did you enjoy playing this game?” and the child gave their answer, either pointing or touching the smiley, or verbally. The answers were recorded by the therapists at the end of every session.

To collect other useful information related to each session, the following tools were designed and developed:

- *Therapist’s diary*: at the end of every session, therapists wrote notes regarding the most significant and interesting aspects of the experience. These annotations were analysed to integrate the collected data with qualitative observations.
- *Application log*: the games were designed using a software framework which allows the collection of a variety of technical information, including the positions of the child and of the interactive elements, the actions taken over time, and other contextual information from the game. These logs will be analysed to provide objective reaction times and as an integration to reinforce the analysis of other data sources.
- *Video recording*: a camera recorded all the sessions to provide an objective and detailed record of the progress of the child. These videos will be analysed exclusively by the authorized personnel in order to protect the privacy of the subjects. This analysis will provide additional information about the reaction time, confirming the measurements provided by the logs, and will also allow to evaluate the child’s experience from an external and expert point of view. Data on training performances was collected on a remote database available at the RHF.

The usability of the system was investigated using a questionnaire created and administered to all the four therapists involved, after the conclusion of the training. The questionnaire was composed of nine questions (Q1-9) on the experience of using the mini-games, followed by the ten standard questions (Q10.1-10) of the Systems Usability Scale (SUS) questionnaire [7], all of these on a 5-point Likert scale. In addition, each question in the first part allowed the respondents to add comments, and further comments were encouraged after the SUS section.

### Method

Children played these digital games at the RHF for 5 consecutive weeks, once a week, for 15 minutes each session, in the period of November-December 2021. Every child was introduced to the proposal by the referral therapist. The therapists involved were trained in the use of the system and in how to propose the games to the children. The starting and ending game of each session was the same for all participants for the overall program. The intermediate games were chosen, when possible, together with the child, and according to their preference. The referral therapist was present during all the sessions. When necessary, a second therapist was involved to manage the computer system, in order to help the other therapist to focus entirely on the child.

Every session began and ended with a game of *Bubbles*, the same for all the children and for the whole period of training. Specifically, the game was configured to present a single black bubble on a white background, with slow moving speed, maximum size, one bubble at a time. The game ended when the child burst the tenth bubble. Each child started the game in the same position. The intermediate proposal was tailor-made by the therapist in relation to the preference of the child, the rehabilitative objectives of the training, and the performance of the child during the previous session. The intermediate proposal could be either a game of *Bubbles* (with different characteristics), *Sound explorer*, or *Ping pong*. Whenever possible, the therapist stayed outside the play area.

### Results

#### Testing the children’s satisfaction

The children expressed very high satisfaction regarding the games. Almost all of them gave the maximum score (3) for all the sessions (see Table 2). Some children spontaneously requested to play with them every week. Several of them would have liked to continue even after the conclusion of this test session. Most of them expressed their satisfaction even during the game (“It is magic!”, “I can’t wait to burst the bubbles”, “Teacher, this amuses me!”). The children generally showed greater motivation in the first few sessions, while the interest dropped at the third or fourth match for some of them. All the children understood the test question regarding satisfaction, except one. For this child, the question was “Did you enjoy playing this game?” and he answered “yes” every time.

**Table 2:** Levels of Satisfaction expressed by children using the tactile VAS Scale

Session	Score 1	Score 2	Score 3
	Not at all	So and so	Very much
1^st^	0	1	6
2^nd^	0	0	5
3^rd^	0	0	5
4^th^	0	0	7
5^th^	0	0	5

#### Usability questionnaire

We collected responses to a usability questionnaire from the four therapists involved in the study. The questionnaire is available in Italian^4^ and the following analysis refers to the question numbers. The Likert scale results are summarised in Table 3.

**Table 3:** Results of the usability questionnaire (Section 4.4.2)

	1	2	3	4	5
(a) Usage experience questionnaire					
**Q1**	**2**	**2**	0	0	0
**Q2**	0	0	1	**3**	0
**Q3**	**2**	1	1	0	0
**Q4**	0	1	1	**2**	0
**Q5**	0	0	1	**2**	1
**Q6**	0	0	1	**2**	1
**Q7**	1	**2**	1	0	0
**Q8**	0	0	**2**	**2**	0
**Q9**	0	0	0	**2**	**2**
(b) SUS questionnaire					
**Q10.1**	0	1	**2**	1	0
**Q10.2**	**3**	1	0	0	0
**Q10.3**	0	1	**2**	1	0
**Q10.4**	**3**	1	0	0	0
**Q10.5**	0	**2**	**2**	0	0
**Q10.6**	**2**	0	**2**	0	0
**Q10.7**	0	0	**3**	1	0
**Q10.8**	**3**	0	1	0	0
**Q10.9**	0	0	**4**	0	0
**Q10.10**	**3**	1	0	0	0

The column headings correspond to a 5-point Likert scale going from Strongly Disagree (1) to Strongly Agree (5). The most frequently-chosen answers are highlighted in bold

Overall, the usage experience was positive, although some technical issues emerged. The therapists found that the gameplay instructions were easy to understand (Q1) and relatively easy to explain to the children (Q6), depending on each child’s condition. One respondent wished there was a “standard” explanation offered to all the therapists, and clearer instructions as to whether the therapist is allowed to verbally intervene during play. All the respondents felt comfortable using the system by themselves (Q9), but also thought that they could sometimes benefit from the presence of a colleague during the sessions (Q4), to help with the technical aspects. Some respondents noted that such benefit would very much vary from child to child, where some children picked up the system so quickly that they could even set the games running by themselves, whereas others would be inhibited by the presence of others to the point of being unable to engage with the therapist and the games.

All the therapists found that the mini-games helped them to improve their therapy work (Q5) with the ability to quickly change the games’ configuration and test each child’s ability and progress. The mini-games were also found to help focus on new aspects of the therapy work (Q2), particularly by helping to strengthen the relationship with the children, and by allowing the therapists to observe how the children observe, explore, and interact with their surrounding space. One therapist observed that some children find it difficult to observe and react to obstacles and moving objects, such as a ball, a car, or another child. They observed that the presentation of visual stimuli that do not present a threat to the child may help them focus their attention on their surroundings and react accordingly. Overall, the mini-games were not considered a hindrance to therapy (Q7) although suggestions were made to develop more games, and to include skills progression within the games.

With the exception of two children, the system did not create a barrier between the therapists and the children (Q3), instead of helping to create and strengthen the relationship (Q8). The two exceptions comprised one child who sternly refused to participate due to the sessions being video-recorded, and one child who would not step inside the play area, although the therapist notes that this child was possibly too young to begin with.

The four responses to the SUS questionnaire (Q10.1-10) scored between 50 and 62.5, with an average of 56.25, corresponding to a “marginally acceptable” usability, according to previous research [5].

Four respondents is a small sample even by the standards of the SUS questionnaire which is often applied to at least eight to twelve participants [7]. It is also worthwhile noting that some of the questions may not necessarily apply to our system, so the responses may only be trusted up to a point. Nevertheless, it is clear from the comments collected that there are opportunities for improvement and the system was well received and liked.

## Discussion

The following sections are the results of the discussion within the co-design team, including both therapists and computer engineers (see Section 3). The discussion moved from the the data acquired with the surveys, the observation of the video recordings, and some spontaneous comments by the therapists, collected after having tried the mini-games with the children.

### Adaptation of the games

The possibility to select the characteristics of the game through an intuitively usable interface allowed the therapists to create a suitable proposal for each child in that specific moment of their growth. Specifically, the games were adapted in relation to the wishes of the child, to their level of activity or tiredness, and to the specific rehabilitation objectives. The use of high colour contrast, large size, and low speed bubbles put all the children in the best conditions to express themselves, including those with the lowest visual residue. This factor was decisive in supporting motivation and adherence to the proposal. In addition, the possibility to read the game’s commands (i.e., colour, size, speed, etc.) directly on the floor projection, thanks to large, capital letters, directly involved the children in the customization of the games, modifying the parameters together. In this way, it was possible to co-design the training program, contributing greatly to increase the children’s motivation, intent, and active involvement in the game, and to making the game really adapted and accessible for them at the same time.

Most of the children requested “big” and “slow” bubbles for the first sessions, while toward the third session they opted for smaller and faster bubbles. An initial phase of adaptation appeared important to become familiar with the game. In fact, the novelty initially made some children fearful, even if they appeared intrigued. However, after an initial moment of greater caution, all of the children except one expressed high satisfaction and concluded the program.

### Preparation of the children to the games

This “test experience” has highlighted how not only it is important to create adapted games, but it is equally significant to plan the way to prepare and accompany the children through the game. It is crucial to create a context that makes the children feel at ease and comfortable in order to help them to better express their skills. In fact, some children appeared hesitant and frightened in the first session. This behaviour has been observed in particular in children with lower visual residue, and especially when they had no previous experience of the room, and entered directly in an environment that was already set up – i.e., dark room, projector on.

Therefore, it seemed essential to spend some time before the start of each session during which the children would be guided to explore the room, which is new to the majority of them, as it is in normal conditions, and then to gradually create the ideal environment together for using the interactive floor – i.e., pulling the curtains, turning the projector and the PC on, turning the room’s lights off, and so on. The children themselves often asked how the system worked, and on the conditions of its functioning – i.e., “Why do you have to turn off the light? Where does it turn on? Where does the light come from?”. They felt gratified for being the ones who turned on the game – “the magic wand makes everything more magical”, said one child while he turned on the projector with the remote control. Furthermore, the verbal anticipation of sudden noises and the preliminary experience of the game in the test screen allowed the children to prepare themselves for the new situation. Returning together to the initial environmental condition at the end of each session – i.e., turning off the projector and turning the room’s lights on – allowed the children to mentally conclude the game session. The creation of a space-time containment is important to make the children feel safe and therefore to be able to enjoy the games with more initiative. Furthermore, the presence of a known reference figure nearby, especially in the early stages of the experience and, when necessary, with verbal and/or physical mediation during the game, also appeared to be significant. Two small children in their first session asked to play the game in the therapist’s arms (“I want to do this in your arms”). For these children, the presence of a second person in the room for the PC management appeared important so that the therapist could focus completely to accompanying the child into the game. Verbal anticipation and doing things together can be two useful strategies to prepare children to the novelty, and to support their well-being and motivation. With some children there was a need for dilated time to adapt to both the setting and the game.

**Fig. 9:**
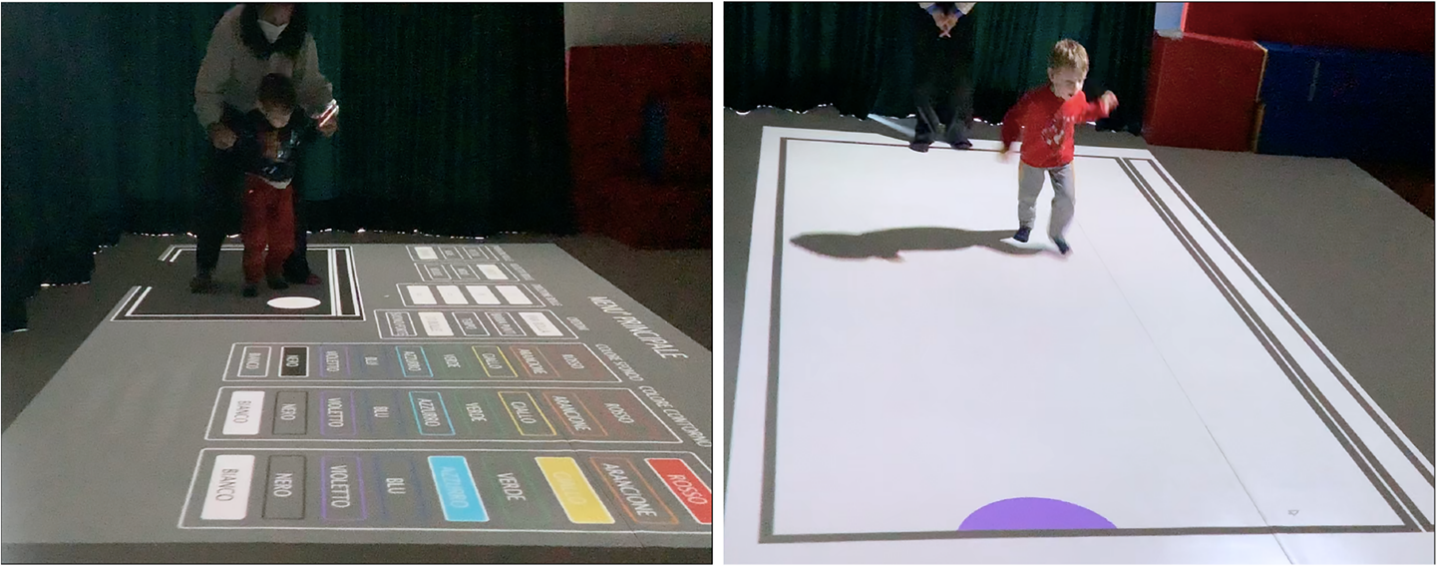
In the left image, a child in the first session of the play, searching for a reassuring contact with the therapist. On the right, the same child in the second session, playing independently

For these reasons, therapists had to think and to adapt the way of presenting these games to the children in order to better prepare them to live this experience with serenity (see Fig. 9).

### Progression and customization of the game sessions

This experience with the children allowed the professionals to better understand how the children perceive the reality around them if placed in the most facilitating and adapted conditions. In particular, it made it possible to observe the following skills of the children:

- ability to adapt to novelty, and time needed to feel at ease;
- use of vision to explore the surrounding space (i.e., neglected area, reaction time, perception and localization of target etc.);
- use of oculomotor skills (i.e., fixation, saccadic movements, smooth pursuit) in order to orientate themselves and navigate in space;
- ability to maintain, shift, and share attention (i.e., reaction time, visual crowding etc.);
- online visual monitoring of movement (i.e., quality of visual-motor coordination, movement accuracy etc.);
- ability to orientate in space (i.e., internalization of topological concepts etc.);
- motion perception;
- colour recognition;
- compliance with waiting turns and social rules.

Furthermore, the qualitative, in-loco observation, as recorded by the therapists, and the analysis of the video recordings, made it possible to identify some critical areas in child’s development and skills, such as difficulties in respecting the turns in the game, missing or altered recognition of some colours, forgotten areas in space exploration, or difficulties in orientation in a crowded environment – e.g., with multiple bubbles at the same time. These observations represented the basis for the definition of the rehabilitation objectives: these were defined by therapists during the first session and gradually revised in relations to the modification of parameters of the games and of the children’s behaviour.

**Fig. 10:**
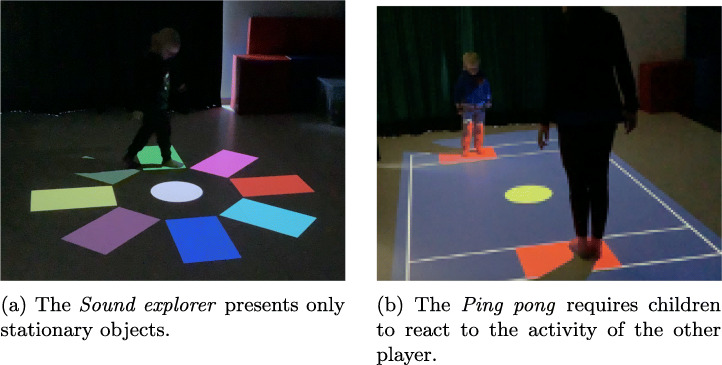
Progression of complexity of games

The possibility to customize the proposals with increasing levels of difficulty (i.e., increasing the speed and the number of bubbles present simultaneously, and/or reducing the colour contrast) has not only allowed us to understand which conditions promote the best performance of the children, but also to identify their critical areas (see Fig. 10). It is important to intervene on these through a personalized rehabilitation program in order to support the children in transferring skills to the real world.

### Guidelines

These guidelines are the result of the discussions within the multidisciplinary team that took place during the design thinking process and after having evaluated different prototypes and setup configurations. They are aimed at designers interested in developing games for children with VI using large-scale interactive environments.

- The background has to be as uniform as possible, to easily identify the foreground objects;
- The background colour has to be configurable to guarantee an optimized contrast, in relation to the visual ability of the child with VI;
- The foreground objects have to be simple shapes, at least initially. More complex and detailed shapes can be introduced in a second phase, for children that show improved abilities to recognize objects;
- The objects colour has to be configurable for each user, given quite common personal differences in colour perception;
- The smallest object should have a dimension of at least 10 cm, but the correct measure should be configurable in relation to the need of the child;
- The maximum velocity of a moving object has to be 1 m/s. Velocity has to be configurable in relation to the visuo-motor ability of the child;
- Lines must have a thickness of at least 5 cm (for example, for delimiting the playground).

It is important that therapists follow specific precautions so that the system can be easily accepted by the children, and to encourage the most functional use of these games. In this way, the child is allowed to face the new proposal with greater serenity. The precautions to be followed are listed here:

- The caregiver has to verbally explain the characteristics of the game to the children in advance, to help them to prepare for the new game;
- The children have to be guided to explore the physical environment in normal conditions (for example, room’s light on, PC and projector turned off) before the game, in order to be able to better orient themselves in the play area;
- Suitable environmental conditions for the use of the system have to be created, if possible, together with the children (i.e., turning off the light, pulling the curtains, turning on the video projector) to make them feel more confident and involved;
- The sudden arrival of sounds has to be verbally anticipated, in order to not frighten the children;
- If possible, the children have to choose the game and its characteristic (i.e., size and speed of the bubbles) together with the therapist, to make the game tailor-made to them;
- The therapist has to stop on the configuration interface for a time necessary for the children to understand the functioning of the game and to feel more confident with the proposal, guiding them verbally or physically;
- During the game, the therapist has to stand in a fixed position and give verbal feedback to the children in order to show their presence and location in the environment, helping them to feel more secure and to better orient themselves in space;
- It is important to allow some time for the children to ask questions or clarify doubts about the game, its characteristics, and the functioning of the system, to make them feel more involved.

## Conclusions

Following a design thinking approach, a set of mini-games were designed for children with visual impairment. To address the requirements, the user interface for the games is based on a large-scale interactive environment. The usability and the appraisal of the mini-games have been evaluated by means of a pilot experiment in a real therapeutic setup. The results provided us with suggestions on how to improve the system and have allowed us to collect some guidelines of general interest for those who want to develop applications for children with VI, using large-scale interactive environments. Following the comments of the therapists involved in the experiment, the interactive environment developed at the RHF can be a useful tool to support the engagement and motivation of the children, and to promote the achievement of rehabilitation goals. This experience has also highlighted how important it is not only that games must have adapted characteristics for children with VI, but also that the modality of proposal inside the therapeutic path has to be tailor-made to individual peculiarities.

## Electronic supplementary material

Below is the link to the electronic supplementary material.

(mp4 5.22 MB)

## Data Availability

The datasets generated and analysed during the current study are available from the corresponding author on reasonable request.

## Footnotes

1 More tools and good practices to improve the accessibility of digital games con be found at https://ablegamers.organdhttps://accessible.games.

2 The term mini-game is used in this paper to emphasise that the three digital games were designed with basic game mechanics (one level and a simple scoring mechanism) to render them suitable to the users and the activities of the Hollman Foundation, where each rehabilitation session has a limited time duration.

3 https://processing.org

4 http://www.dei.unipd.it/~roda/mini-games/questionnaire.pdf
